# Vaccines and Monoclonal Antibodies as Alternative Strategies to Antibiotics to Fight Antimicrobial Resistance

**DOI:** 10.3390/ijms25105487

**Published:** 2024-05-17

**Authors:** Chiara La Guidara, Roberto Adamo, Claudia Sala, Francesca Micoli

**Affiliations:** 1 Magnetic Resonance Center CERM, University of Florence, 50019 Florence, Italy; 2 Department of Chemistry “Ugo Schiff”, University of Florence, 50019 Florence, Italy; 3 GSK, 53100 Siena, Italy; 4 Monoclonal Antibody Discovery Laboratory, Fondazione Toscana Life Sciences, 53100 Siena, Italy; 5 GSK Vaccines Institute for Global Health S.R.L. (GVGH), 53100 Siena, Italy

**Keywords:** antimicrobial resistance, vaccines, monoclonal antibodies, antibiotics, innovative technologies, mode of action

## Abstract

Antimicrobial resistance (AMR) is one of the most critical threats to global public health in the 21st century, causing a large number of deaths every year in both high-income and low- and middle-income countries. Vaccines and monoclonal antibodies can be exploited to prevent and treat diseases caused by AMR pathogens, thereby reducing antibiotic use and decreasing selective pressure that favors the emergence of resistant strains. Here, differences in the mechanism of action and resistance of vaccines and monoclonal antibodies compared to antibiotics are discussed. The state of the art for vaccine technologies and monoclonal antibodies are reviewed, with a particular focus on approaches validated in clinical studies. By underscoring the scope and limitations of the different emerging technologies, this review points out the complementary of vaccines and monoclonal antibodies in fighting AMR. Gaps in antigen discovery for some pathogens, as well as challenges associated with the clinical development of these therapies against AMR pathogens, are highlighted.

## 1. AMR: A Global Threat to Public Health

The introduction of antibiotics into clinical use is considered one of the major medical breakthroughs of the 20th century, as it completely revolutionized the treatment of bacterial infections and significantly improved the health of many patients worldwide [1]. In 1928, the discovery of penicillin by Alexander Fleming started the so-called “golden age of antibiotic discovery”, which reached its peak in the 1950s [1]. Since then, the overuse and misuse of antibiotics in humans and animals has resulted in the selection of drug-resistant pathogens giving rise to the current AMR crisis [1].

AMR is silently evolving as the next potential pandemic, and it has been estimated that the number of deaths due to AMR will exceed those caused by cancer by 2050 [2]. AMR occurs when microorganisms (e.g., bacteria, viruses, fungi, parasites) accumulate mutations over time and no longer respond to antimicrobial drugs, which become less effective or ineffective [3]. This complicates the treatment of infections and the risk of disease spreading, with a great increase in severe illness and mortality.

Although AMR is an evolutionary phenomenon that occurs naturally in microbes, usually using genetic changes, the inappropriate use of antibiotics in humans and animals constitutes a key driver to its spread [4]. Global antibiotic consumption has increased by 65% from 2000 to 2015, mainly in low- and middle-income countries (LMICs), and is projected to triple by 2030 without appropriate interventions [5].

Over the last few years, the World Health Organization (WHO) and numerous other authorities called for coordinated action to address AMR. In 2015, with the Global Action Plan (GAP), the WHO highlighted the need for a “One Health approach”, i.e., the joint action in human health, food production, animals and environmental sectors, to achieve better public health results [6]. In the same year, the WHO initiated the Global Antimicrobial Resistance and Use Surveillance System (GLASS), a standardized approach to collect, analyze and share AMR data coming from all countries [7].

To guide and support research and development of new antibiotics, the WHO published a list of antibiotic-resistant “priority pathogens” in 2017, where bacteria belonging to 12 families were classified as critical, high or medium priority, according to the urgency for new antibiotics [8]. Another fundamental step to strengthen the fight against AMR was achieved in 2019, when the AMR Multi-partner Trust Fund (AMR MPTF) was launched to intensify efforts to support countries, especially the LMICs, to face the AMR threat [9]. More recently, the WHO underlined the need to maximize the use of existing vaccines and accelerate the development of new ones against AMR pathogens [10] that were categorized according to biological analyses, product development and access and implementation feasibility.

**Figure 1:**
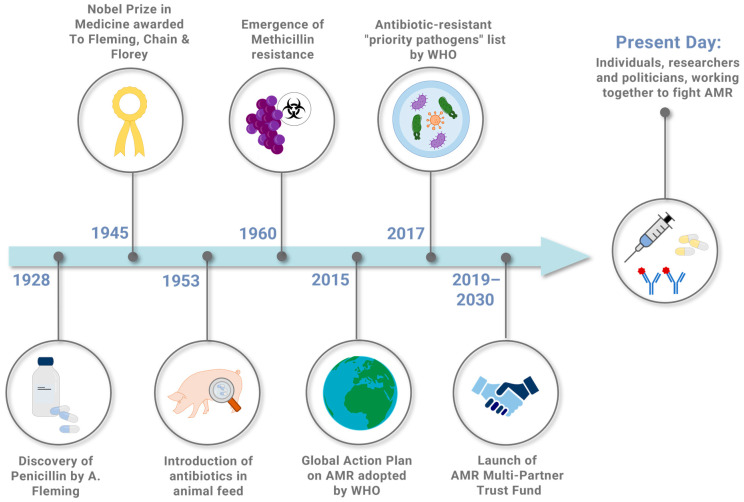
Timeline showing some key milestones, among the most important steps, in the history of antibiotics and antimicrobial resistance.

Nowadays, a significant effort is exerted by the scientific community engaged in developing new alternative therapies to traditional antibiotics. Figure 1 summarizes the most important steps in the history of antibiotics and antimicrobial resistance.

AMR is responsible for many deaths every year, affecting both high-income countries (HICs) and LMICs. According to the Global Burden of Disease 2019 report (GBD 2019), 4.95 million deaths are associated with bacterial AMR, including 1.27 million deaths attributable to AMR, with remarkable prevalence in sub-Saharan Africa and South Asia [11]. Data underline that AMR also affects the world’s poorest countries [11]. Several factors facilitate the rapid spread of AMR in LMICs, such as uncertain access to safe water and proper hygiene conditions, quality healthcare systems restricted to a minor part of the population and improper use of antibiotics in humans, animals and crops [12]. Additionally, unlike HICs, where the AMR emergency is periodically monitored by defined surveillance systems, most LMICs lack adequate and standardized programs, making it difficult to estimate the extent of AMR [12].

Among the bacterial pathogens considered in the GBD 2019 study [11], *Escherichia coli*, *Staphylococcus aureus*, *Klebsiella pneumoniae*, *Streptococcus pneumoniae*, *Acinetobacter baumannii*, and *Pseudomonas aeruginosa* were identified as the six leading pathogens, each responsible for more than 250,000 deaths associated with AMR, in agreement with the list of antibiotic-resistant pathogens that the WHO published in 2017 [8]. AMR pathogens associated types of antibiotic resistance and preventive/treatment options under development can be found in Table 1.

## 2. Vaccines and mAbs as Alternatives to Antibiotics in Fighting AMR

While the AMR crisis is spreading worldwide, leading to the emergence of new drug-resistant pathogens, research is exploiting an integrated strategy involving the synergistic action of vaccines, monoclonal antibodies (mAbs), diagnostic tools, microbiota and bacteriophages together with new antimicrobials [40]. Here, we focus our attention on vaccines and mAbs, we highlight the major mechanistic differences with respect to antibiotics and describe the progress of the technologies that support the development of novel products to fight AMR.

Antibiotics can kill bacteria (bactericidal) or stop their growth (bacteriostatic) and are grouped according to their mode of action: (i) inhibitors of cell wall synthesis (e.g., β-lactams and glycopeptides); (ii) cell membrane depolarization compounds (e.g., lipopeptides); (iii) inhibitors of protein synthesis (e.g., aminoglycosides, tetracyclines, oxazolidinones); (iv) inhibitors of nucleic acid synthesis (e.g., quinolones, fluoroquinolones, rifamycins); and (v) inhibitors of metabolic pathways (e.g., sulfonamides) [41].

Bacterial resistance to antibiotics can be natural or acquired [40]: natural resistance occurs when bacteria are resistant a priori, and it may be intrinsic (resistance-associated genes always expressed in the species independently from exposure to antibiotics), or induced (resistance genes are naturally occurring in bacteria, but are only expressed to resistance levels after antibiotic exposure) [40]; acquired resistance occurs by acquisition of genetic material through horizontal gene transfer or by mutations that bacteria accumulate in their genome [38].

Antibiotic resistance is mediated by three main mechanisms:

(i) Resistance mechanisms that prevent access of the antibiotic to its target: bacteria can reduce their outer-membrane permeability (via downregulation of the expression of selective porin proteins), which results in poor penetration of the antibiotics, or can overexpress efflux pumps conferring high levels of resistance.
(ii) Resistance mechanisms involving modification of the antibiotic target and generation of the so-called “escape mutants”: bacteria can change the target’s structure to prevent antibiotic recognition and binding by generation of a mutant of the target through point mutation(s) or recombination; and by post-translational modifications of the target by addition of chemical groups by specific enzymes.
(iii) Resistance mechanisms that inactivate the antibiotic: bacteria can express enzymes that hydrolyze antibiotic molecules, thus preventing binding to the target and resulting in resistance. Also, bacterial enzymes can modify the antibiotic structure by the addition of chemical groups, thereby making the interaction with the target impossible because of steric hindrance. We refer to recent reviews for a better overview of antimicrobial resistance mechanisms and how these have evolved and continue to spread [42,43,44]. A better understanding of the factors contributing to the persistence and emergence of AMR may support the design of new antibiotics from one side and highlight the need for alternatives on the other side.

In marked contrast, the probability of developing resistance mechanisms to vaccines is extremely low [40] and, even in the rare instances in which resistance to vaccines has been reported [45,46], a reduction in disease burden has been achieved anyway. This is an important difference with antibiotics, for which the effect on a patient can be completely abolished by the emergence of resistance. Vaccines are usually administered before bacteria start to multiply and protect the patient from possible infections. On the other hand, antibiotics act therapeutically on ongoing infectious diseases and encounter an increased number of bacteria with a high probability of selecting resistant variants.

**Figure 2:**
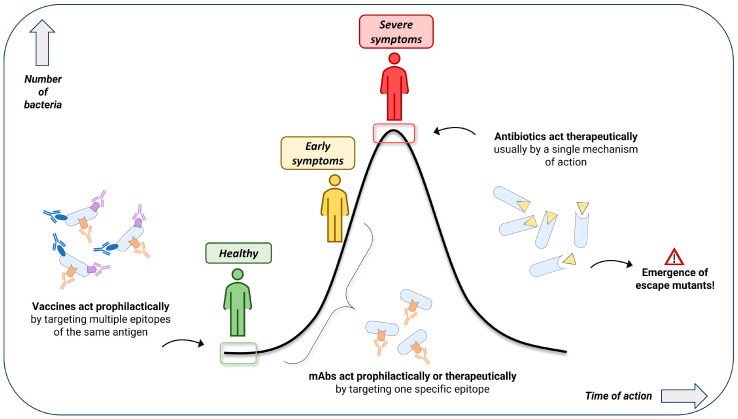
Different modes of action of vaccines, mAbs and antibiotics.

Differently from antibiotics that have a specific bacterial target, vaccines induce immune responses against multiple targets (called antigens) and/or multiple epitopes of the same antigen (polyclonal antibodies) (Figure 2). Consequently, the risk of emergence of vaccine escape mutants is greatly decreased since several mutations would be required for different epitopes [40].

In contrast to antibiotics, which usually inhibit essential bacterial functions, mAbs can bind to essential and non-essential antigens, like virulence factors, and may be associated with a reduced risk of selecting resistant mutants because mutations in these targets may result in reduced virulence and enhanced clearance by the immune system. For example, Tkaczyk and colleagues showed that mutations in the *S. aureus* alpha-toxin epitope targeted by mAb MEDI4893 reduced bacterial fitness upon infection [47]. On the other hand, the extreme specificity of mAbs, especially when they target highly variable antigens such as capsules and O-antigens, makes their spectrum narrow. However, the progress in diagnostics, with the possibility to precisely identify the pathogen responsible for the infection, is likely to help the exploitation of mAbs as novel therapeutics. In addition, mAbs benefit from a naive repertoire as large as 10^12^ possible molecules thus expanding enormously the number of potential candidates to be tested as anti-bacterial medications [48]. Also, mAbs are intrinsically safe as they are bioproducts, and little or no toxicity has been reported so far in clinical trials [49], while small molecules, though less expensive than mAbs, are associated with augmented risk of side effects.

The advantages and disadvantages of traditional antibiotics and their main therapeutic alternatives are summarized in Table 2.

## 3. Innovative Vaccine Technologies to Fight AMR

**Figure 3:**
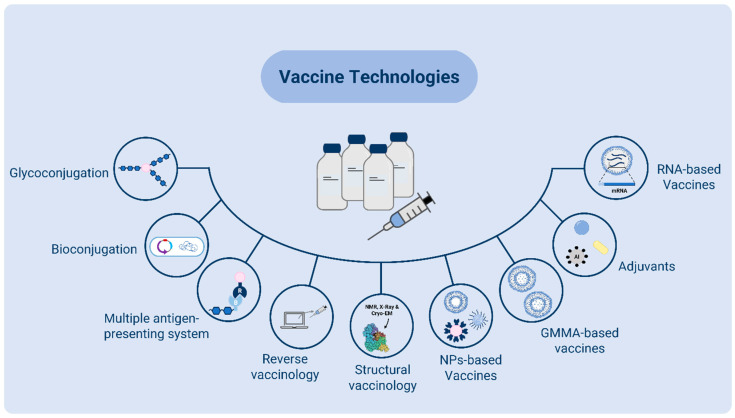
Different innovative technologies are available and can be exploited to accelerate the development of vaccines against AMR pathogens.

Currently licensed vaccines are prepared from live bacteria and viruses by inactivation, attenuation or purification of immunogenic components (subunit vaccines). Over the last few years, scientific progress in many areas has supported the advancement of many technologies for vaccine design and manufacturing [40] (Figure 3).

Glycoconjugation: It is based on the covalent linkage of a bacterial polysaccharide obtained from a natural source to a carrier protein (typically the genetically modified diphtheria toxoid CRM197, or chemically detoxified tetanus and diphtheria toxins). The protein is needed to provide T cell help to carbohydrates that are T cell-independent antigens, thus producing IgM to IgG switch and a memory response. Examples of glycoconjugate vaccines licensed worldwide are the ones against *Haemophilus influenzae* type b, *Neisseria meningitidis* serogroup A, C and ACWY, pneumococcal vaccines, which have been expanded from 7 to 20 valent, and *Salmonella enterica* subsp. enterica serovar Typhi [50]. Continuous broadening of pneumococcal vaccine to cover new strains is needed to overcome serotype replacement and counteract antimicrobial resistance [51]. Bivalent vaccines combining *S.* Typhi and *S.* Paratyphi A O-antigen (O:2) conjugates have entered clinical trials [39]. Investigational glycoconjugate vaccines against *Shigella spp* are also under development (Table 1) [52].

Bioconjugation: Recently, a process to generate glycoconjugates in vivo has been developed by exploiting the oligosaccharyltransferase PglB from *Campylobacter jejuni*, which exhibits a relaxed substrate specificity towards glycans and allows the transfer, in *E. coli,* of heterologous polysaccharides to carrier proteins containing specific N-glycosylation sequences. By incorporating in *E. coli* the glycan operon, along with the oligosaccharyltransferase PglB and the protein-encoding plasmid, it is possible to produce in a single fermentation step the glycoprotein, thus simplifying vaccine manufacturing and preserving the protein epitopes in their natural conformation. Bioconjugates for the prevention of different AMR pathogens, including *Shigella*, *S. aureus*, *P. aeruginosa* and *K. pneumoniae*, are under development and some candidates have reached the clinical phase (Table 1) [50,53,54]. The most advanced one is a 9-valent vaccine targeting invasive infections caused by Extraintestinal Pathogenic *E. coli* (ExPEC-9V) [20]. Bioconjugates composed of capsular polysaccharides from *K. pneumoniae* based on the oligotransferase PglS which glycosylates O-serine residues of the protein carrier have been also developed [55]. Based on this approach, a heptavalent vaccine candidate including O-antigen and capsular polysaccharide conjugates has been obtained [56].

Multiple Antigen-Presenting System (MAPS): A MAPS is based on the formation of multivalent immune complexes, containing both polysaccharide and protein antigens mimicking features of a whole-cell construct for B and T cell activation [57,58]. To obtain the MAPS complex, a target protein antigen is genetically fused to an avidin moiety (termed rhavi) and incubated with a biotinylated PS [58]. This design appears advantageous to target challenging AMR pathogens, such as *S. aureus*, a bacterium characterized by complex host–pathogen interactions and a variety of escape mechanisms [59]. Depending on the type and size of the glycan–protein complex, the MAPS has been shown to drive Th1/Th17-biased immune responses [58]. Current MAPS vaccines under clinical development include the 24-valent pneumococcal vaccine that was successful in Phase 2 clinical studies [60]. A multivalent vaccine targeting invasive *K. pneumoniae* and *P. aeruginosa* infections is also in development.

Reverse vaccinology: This technology enables the selection of potential vaccine candidates by sequencing the bacterial complete genome to select, screen and test the encoded surface-exposed proteins as vaccine candidates in both in vitro and in vivo preclinical models. This approach has been successfully applied to the design of the meningococcus B vaccine [61] and has been exploited for the identification of vaccine candidates against *Shigella* [62], *P. aeruginosa* [63] and *Enterococci* [64].

Structural vaccinology: Isolation of protective human mAbs from convalescent sera and structural studies on antigen–antibody complexes have been combined to design more potent antigens [57]. Although very recent, this technology has already delivered a vaccine to combat respiratory syncytial virus (RSV) where the F protein of the RSV responsible for viral entry has been stabilized in the immunogenically relevant prefusion conformation [65]. Structural vaccinology has been used to inform the design of vaccines against HIV and to develop universal vaccines against influenza and, more recently, COVID-19. Human mAbs have allowed the identification of novel protective antigens, such as the CMV pentameric complex [66]. Of note, structural changes can be relevant also for bacterial proteins; therefore, this approach can be used to ensure the proper conformational presentation of these proteins or to select cross-protective antigens against different bacterial strains or serotypes [67].

Nanoparticle vaccines: Further advances in protein engineering have allowed the highly ordered display of epitopes in various nano-sized scaffolds [68]. This type of particle has the potential to promote antigen location in lymph nodes and enhance antigen uptake by antigen-presenting cells and B-cell receptor cross-linking as compared to subunit protein antigens [69]. Self-assembling viral structural proteins are already used in commercial vaccines targeting the hepatitis B virus, the human papillomavirus and the hepatitis E virus [70,71]. Today progress in the in silico protein design has enabled the optimization of protein density and the formation of multimers in the correct conformation [72]. The resulting protein nanoparticles can be more immunogenic compared with soluble recombinant proteins and can be combined with novel adjuvants to obtain optimal antibody titers. An alternative approach for nanoparticle vaccines is the SpyTag–SpyCatcher technology, which is based on the spontaneous formation of stable isopeptide bonds between a SpyCatcher genetically encoded protein and its peptide-partner SpyTag. This approach allowed for the decoration of virus-like particles with a single antigen, such as the malaria protein Pfs25 [73] or twin antigens, including, for instance, Pfs25 and Pfs28 [74]. The SpyCatcher/SpyTag protein ligation technology has been also exploited to link antigens to *E. coli* virulence factor hemoglobin protease that can be highly expressed on outer membrane vesicles (OMVs). Through this approach, OMVs exposing the pneumococcal antigens PspAα have been built, demonstrating applicability to AMR pathogens [75].

Generalized modules for membrane antigens (GMMAs): GMMAs are OMVs produced by Gram-negative bacterial strains that have been genetically modified to enhance their release. GMMAs, and more in general OMVs, are advantageous to favor the presentation of antigens in their natural environment and conformation and promote uptake by immune cells because of their nanosize. Also, the presence on the surface of lipoproteins and lipopolysaccharide (LPS) exerts an immune stimulatory effect that enhances immunogenicity. Typically, the lipid A portion of GMMA LPS needs genetic modification to modulate endotoxicity [76]. A 4-component GMMA-based vaccine against *Shigella* is currently in Phase 2 [77,78] and a bivalent formulation of *S.* Typhimurium and *S.* Enteritidis GMMAs (NCT05480800), and its combination with a glycoconjugate vaccine against *S.* Typhi (Typhibev) (NCT 05480800), have recently entered the clinical phase. OMV-based vaccines against *Neisseria gonorrhoeae* are under investigation [79,80,81] and the GMMA approach has been exploited to develop a vaccine candidate in the clinical phase (NCT05630859).

Additional engineering can be applied to incorporate heterologous polysaccharides on the LPS backbone resulting in glycan-decorated GMMAs. Through this approach, glycosylated OMVs exposing pneumococcal capsular polysaccharides [82] or *S. aureus* poly-N-acetylglucosamine (PNAG) have been obtained, showing promising results in preclinical models [83]. Chemical conjugation of a variety of glycan and protein antigens has been demonstrated feasible and shown to enhance the immune response against the target antigens in animal models [84].

Adjuvants: Adjuvants are used to enhance the immune response elicited by subunit vaccines. Currently, a variety of adjuvants, such as AS01, AS03, MF59, AS04, CpG and other Toll-like receptor agonists, are licensed [85]. AS01 is a liposomal formulation of the saponin QS21 and monophosphoryl lipid and has been licensed for a vaccine against malaria, herpes zoster and, more recently, RSV [86,87,88]. The AS01 adjuvant has also been used in an investigational subunit vaccine designed to prevent reactivation of tuberculosis, which showed 54% efficacy in clinical studies [89]. Oil water emulsions such as AS03 and MF59 have been used to adjuvant the pandemic flu vaccine to achieve dose sparing along with an increased cross-strain coverage [90] and, more recently, AS03 has been tested in a Phase 1 study in combination with a tetravalent *K. pneumoniae* bioconjugate vaccine (NCT04959344).

RNA: RNA-based vaccines exploit the host cell translational machinery to produce the selected antigens and proved to be game changers to fight COVID-19 [91,92]. Although this technology has been mostly applied to viral pathogens, a self-amplifying RNA vaccine encoding for a double mutant of Streptolysin-O (SLOdm) and the backbone protein of pilus island 2a (BP-2a) from Group A and Group B *Streptococci* was shown to induce both humoral and cellular immunity in mice [93]. More recently, a self-amplifying RNA vaccine that targets *Plasmodium falciparum* macrophage migration inhibitory factor, which is secreted by the parasite and diminishes the host inflammatory response against infection, was shown to protect from malaria reinfections in a preclinical model [94]. The fast manufacturing process and relatively low production cost support the use of this platform for vaccines against AMR infections. The impact of glycosylation that would occur during the bacterial protein expression should be taken into account, as this could mask relevant epitopes or generate unwanted neoepitopes.

## 4. Recent Advancements in mAbs

Human mAbs are among the most transformative and effective medications against cancer and autoimmune diseases [95]. Nonetheless, they are rarely employed for the treatment of infections caused by pathogenic bacteria or viruses. The recent COVID-19 pandemic highlighted the relevance of these innovative drugs, with several candidate mAbs discovered and approved for emergency use at a pace never seen before [96]. In the quest for novel approaches against the spread of AMR, mAbs can play a major role since they bypass the antibiotic mechanisms of action/resistance, are intrinsically safe and frequently represent the only therapeutic option for immunocompromised patients. They can be easily mass-produced and benefit from the enormous technological progress reported in recent years. Finally, mAbs specifically target pathogenic bacteria, thus sparing the microbiota.

Despite the advantages listed above, only a few antibacterial mAbs have succeeded in clinical trials, and, to date, three of them have obtained approval from the FDA: Raxibacumab and Obiltoxaximab for the treatment of inhalational anthrax [97] and Bezlotoxumab for preventing recurrent *Clostridium difficile* infections [98]. Additional mAbs are currently in the discovery or preclinical stage (Table 1). Examples are represented by monoclonals targeting bacteria enclosed in the WHO priority pathogen list, such as *P. aeruginosa* Type 3 Secretion System (T3SS) [16], *N. gonorrhoeae* lipooligosaccharide [32], *K. pneumoniae* O-Antigen and capsule [21,22,99]. All of these bacteria are characterized by a steady increase in antimicrobial resistance rates and some are even classified as pan-drug resistant [100]. In this context, mAbs can offer a therapeutic alternative that goes beyond AMR.

Antibodies, also known as immunoglobulins (Ig), are 150 kDa Y-shaped proteins produced by B lymphocytes. They are composed of two identical light chains, either k (kappa) or l (lambda), and two identical heavy chains which determine the isotype: IgA, IgD, IgE, IgG and IgM. IgG are the most abundant antibodies in human serum and can be further classified into four subclasses, IgG1, IgG2, IgG3 and IgG4, each of them with specific features [101]. IgGs exist as monomers and are the most common molecules for therapeutics thanks to their long serum half-life (21 days), ability to fix complement (especially IgG1 and IgG3) and regulatory effector functions mediated by the Fragment Crystallizable (Fc) portion [102]. IgGs consist of two Fragment Antigen-Binding domains (Fabs) linked to an Fc through a flexible hinge region that confers to Fabs a high degree of conformational flexibility. The two Fabs comprise a variable domain composed of a pair of variable domains, one from the heavy and one from the light chain. The complementarity-determining region of the variable chains defines the Fab antigen-binding site for the mAb to a specific epitope on the antigen [103]. On the other hand, IgM- and IgE-isotype antibodies are in clinical trials, although their half-life is shorter than that of IgGs (3 to 5 days) [104,105], whereas IgA molecules have not reached the clinical stage yet despite their relevance in mucosal immunology [106,107,108].

Developing mAbs is currently possible thanks to the availability of several methods. Hybridomas were first generated in 1975 and consist of combining antibody-producing murine B cells and myeloma cells [109]. Novel methodologies have emerged during the years for cloning and expression of fully human mAbs and these include phage display libraries [108], transgenic mice [109], mammalian expression cell lines and the isolation of mAbs from human B lymphocytes obtained from convalescent or vaccinated individuals. The latter approach is frequently referred to as Reverse Vaccinology 2.0 [110] since it exploits human mAbs as baits for fishing their cognate antigens, which, in turn, can become part of a rationally designed vaccine.

Finally, the most recent advancements in omics technologies and systems biology have allowed the prediction of antigens and epitopes exposed on the bacterial surface that can be targeted by mAbs [111,112].

## 5. Vaccines and mAbs Mode of Action

**Figure 4:**
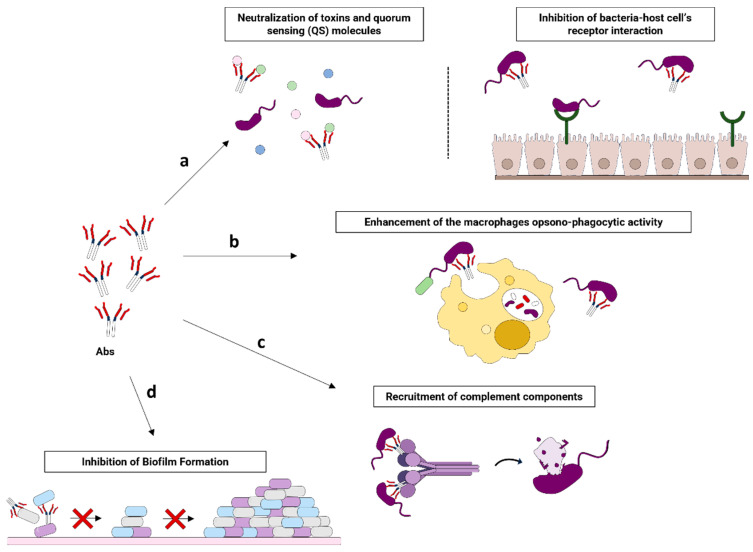
Modes of action of antibodies (either recombinantly produced mAbs or polyclonal antibodies elicited through vaccination). (**a**) Antibodies can neutralize toxins produced by pathogenic bacteria, thus blocking the infectious process; (**b**) antibodies can enhance the opsonophagocytic activity of macrophages and promote elimination of internalized bacteria; (**c**) antibodies can recruit the C1q complement component and initiate the complement cascade, which results in bacterial membrane lysis by the membrane attack complex; (**d**) antibodies can bind adhesins on the bacterial surface, thereby disrupting the interactions within bacteria or between bacteria and abiotic surfaces or between bacteria and cell receptors, thus interfering with adhesion/invasion of epithelial barriers and biofilm formation.

Vaccines and mAbs represent two sides of the same coin and should not be considered to be mutually exclusive but rather complementary in their mode of action. The adaptive immune response elicited by vaccines is mediated by B cells that produce antibodies (humoral immunity) and by T cells responsible for cellular immunity. For most vaccines, the induction of antibodies is critical to confer protection [113]. However, while vaccines generate immunological memory, train the immune system to recognize external invaders and induce a protective response that includes antibody production, mAbs do not exert any training on the immune system nor do they generate memory. However, mAbs constitute an effective and immediately available shield against infections and are therefore more suitable than vaccines, which require 2–3 weeks to elicit the immune response, for addressing health emergencies. On the other hand, vaccines can confer long-lasting protection whilst mAbs usually have a few months’ half-life (Table 2). Antibodies (either recombinantly produced mAbs or polyclonal antibodies elicited through vaccination) can be directed toward exotoxins or bacterial cell surface components, such as proteins and polysaccharides, and can display a variety of mechanisms of action, depending on the antibody and its cognate antigen (Figure 4).

Neutralization is a process by which antibodies selectively bind to either bacterial surface molecules or toxins generated by bacteria, thereby blocking pathogenesis. For instance, raxibacumab specifically targets PA, the protective antigen toxin of *Bacillus anthracis* [114]. Another example is represented by bezlotoxumab, which binds to *Clostridium difficile* toxin B [98]. The mechanism known as complement-dependent cytotoxicity (CDC) involves activation of the complement cascade that starts with the recruitment of C1q, which triggers a series of events leading to the generation of the membrane attack complexes, which in turn causes perforation of bacterial cell membranes and subsequent lysis. The complement pathway contributes, for example, to the host’s defense against *Salmonella* [115], *Shigella* [116,117] and *N. gonorrhoeae* [118]. For this reason, Fc engineering to facilitate IgG hexamerization has been exploited to enhance complement-dependent bactericidal properties of mAbs. It has been shown that by inserting specific mutations, namely E345K and E430G, in the IgG1 backbone, mAbs form hexamers upon binding to their cognate antigen [119]. Enhanced hexamerization and increased CDC were observed upon engineering a mAb against *N. gonorrhoeae*, where higher engagement levels of C1q and increased bactericidal activity were detected in the presence of the mutagenized mAb compared to its wild-type version [34]. Notably, the mAb carrying the E430G mutation performed well in vivo [34].

Through their Fc portion, antibodies can promote opsonophagocytosis, a process where bacteria are coated by antibodies to enhance their recognition and engulfment by immune cells such as macrophages and neutrophils, thereby facilitating the elimination of bacteria by the immune system. The process is known as antibody-dependent cellular phagocytosis and relies on FcγRIIa’s ability to activate macrophages and increase their phagocytic effects [120,121]. An example of such antibody functionality is represented by a humanized mAb against the anti-opsonic Staphylococcal protein A (SpA). This protein binds to the Fc-domain of most immunoglobulin subclasses and disables the effector functions of antibodies, resulting in B cell expansion and secretion of antibodies with no specificity for *S. aureus* [122]. Mutants lacking the Fc binding property have been tested as vaccine candidates [123,124]. An anti-SpA mAb was shown to block Spa activity, thereby promoting complement-dependent cell-mediated phagocytosis of *S. aureus* in human cord blood [125].

Interestingly, it was demonstrated that opsonophagocytic killing was dependent on galactosylation of the Fc portion [126]. While some bacteria developed intracellular survival mechanisms to evade opsonophagocytic killing, mAbs can exert their protective action through the promotion of neutrophil extracellular traps (NET) release. For example, protection against *K. pneumoniae* infection by IgG1 and IgG3 antibodies was shown to be associated with NET release in vitro and in vivo [127]. Fc-mediated effector functions include antibody-dependent cellular cytotoxicity (ADCC), mediated by the interaction of the Fc domain with the corresponding FcγR. ADCC is mainly caused by Natural Killer cells, neutrophils and eosinophils upon activation of FcγRIIIa [128].

Also, antibodies can inhibit bacterial adhesion to host cells, especially epithelial cells, therefore preventing bacterial invasion and infection. Studies on *Bordetella pertussis* demonstrated how murine mAbs targeting bacterial virulence factors (hemagglutinin (FHA), pertactin (Prn), or fimbriae) inhibited bacterial adhesion to epithelial host cells [129]. More recently, a study by Amerighi and co-workers showed that a mAb against the *S. pneumoniae* RrgA adhesin impeded the colonization of human epithelial cells [130]. Monoclonal antibodies targeting the *K. pneumoniae* fimbrial protein MrkA have been isolated through phage display and hybridoma platforms and shown to elicit strong in vitro and in vivo protections against a multi-drug resistant strain [131]. Induction of anti-adhesive antibodies has been also harnessed to develop a vaccine preventing recurrent urinary tract infections caused by uropathogenic *E. coli*, which adheres to uroepithelial cells through the fimbrial protein FimH [132]. Interestingly, since the protein shifts from an extended low-affinity conformation to a high-affinity compressed form following interactions with the mannosylated receptor on uroepithelial cells [133], mAbs able to intercept the high-affinity form and effectively block adhesion have been identified [134].

Lastly, antibodies can prevent biofilm formation or disrupt existing biofilms by interfering with cell-to-cell adhesion mechanisms and attachment to surfaces. Examples, in this sense, include antibodies directed against *Staphylococcus epidermidis* biofilm matrix components, namely PNAG and the accumulation-associated protein, which were shown to reduce biofilm formation [135,136,137].

## 6. Discussion

Vaccines have an enormous potential impact on AMR by reducing antibiotic utilization and decreasing selective pressure leading to the emergence of resistant strains. For licensed vaccines, such as those against *S. pneumoniae* [138] and *H. influenzae* type B (Hib), the impact on antibiotic-resistant strains has been shown [40]. The continuous broadening of vaccine formulations for *S. pneumoniae,* through the incorporation of new capsular polysaccharides covering more and more strains, is expected to also impact AMR. An arsenal of technologies that allow the design of novel and complex vaccines by combining glycan and protein antigens in the same formulations to target different pathogenic mechanisms, as well as diverse strains, is now available (e.g., bioconjugation, MAPS technology, nanoparticles, GMMA) (Figure 3) and are being tested at the clinical level (Table 1). For the RNA platform, recent clinical data showed a quicker waning immunity compared to classic subunit vaccines; therefore, applicability to bacterial targets will require technological improvements [139].

While new technologies are emerging, identifying a population to prove the clinical efficacy of a vaccine remains a challenge. Some of the AMR pathogens driving infections (e.g., recurrent infections by *E. coli* in the urinary tract [140], severe infections caused by *S. aureus* [141], or individuals undergoing elective surgeries and at risk of *C. difficile* infections [142]) occur either in a community or in a pre-hospitalization setting with an incidence compatible with the execution of clinical studies. Targeting infections in the nosocomial environment, where AMR is a critical problem, remains a challenge for the low relative incidence of infections associated with the single pathogen [143] and because of the need for fast-acting one-dose vaccines.

Importantly, compatibility and cooperation between vaccines and mAbs have been reported in the case of SARS-CoV-2 where antibody feedback and epitope masking mechanisms were shown to increase the breadth of vaccine efficacy [91,144]. An important emerging challenge for bacterial infections, such as staphylococcal ones, is the pre-exposure of most of the targeted patients to the bacterium, which seems to present a pre-existing immunity that those vaccines tested so far fail to shift towards a protective immunity [145,146], and calls for alternative vaccine approaches and formulations. A more profound understanding of the immunology behind these infections is needed to guide vaccine development.

Although vaccines for tuberculosis, a major global burden with high levels of drug resistance, are under clinical evaluation, and a vaccine candidate has shown promising efficacy in a Ph2 study [147], there is a need to develop formulations with a higher impact on the infection.

For other AMR pathogens, such as *A. baumannii* or *E. faecium*, the identification of antigens to prevent their infections is ongoing at the preclinical level and none of the emerging candidates has reached the clinical phase.

Despite the great progress in the field of mAb discovery and development, major challenges remain. These include a choice of the best animal models that recapitulate human disease, selection of target patients for effective and timely administration, and mAb doses. The main explanation for the minor number of approved antibacterial mAbs is the poor correlation between preclinical and clinical study results. Antibodies that are effective in vivo in animal models often show limited protection in clinical trials, highlighting a discrepancy that can be explained by the genetic and immunological differences between human and animal models. For instance, DiGiandomenico and co-workers reported the efficacy of a bispecific antibody against *P. aeruginosa* PcrV protein and PsI exopolysaccharide in mouse and rabbit models of infection [17,148]. Unfortunately, the candidate medication was not effective in preventing pneumonia when tested in *P. aeruginosa*-colonized mechanically ventilated subjects in a Phase 2 clinical trial [18]. For these reasons, to successfully employ mAbs as primary therapies against AMR bacteria, establishing clinically relevant in vitro and in vivo models predictive of success in clinical studies should be considered a priority and certainly demands long-lasting efforts.

One critical aspect related to the exploitation of mAbs as medications is strictly linked to their extreme specificity: mAbs recognize well-defined epitopes of an antigen and this can be a limitation when multiple bacterial serotypes exist such as in *K. pneumoniae* [149] and *S. pneumoniae* [150]. The presence of a thick capsular layer masking potentially conserved protein antigens constitutes an additional challenge. Finally, the intracellular lifestyle of some bacterial pathogens has been frequently thought to pose a barrier to mAb activity as antibodies may not be able to cross cell membranes and carry out their protective/therapeutic functions. However, the discovery of TRIM-21-mediated intracellular restriction of viral and bacterial pathogens by binding to opsonizing antibodies modified this view and strengthened the role of mAbs in intracellular immunity [151,152].

While the above issues are being addressed by the research community, considerable efforts are being made to advance the mAb field. For instance, exploration of mAb-antibiotic synergies has started [148] and interesting findings have paved the way to generating antibody–drug conjugates that exploit mAb specificity for targeted antibiotic delivery as in the case of *P. aeruginosa* [153] and *S. aureus* [154]. Of note, Raxibacumab, the first mAb developed against the *B. anthracis* toxin, was approved for the treatment of inhalational anthrax in combination with appropriate antibiotics [155], thus exemplifying the relevance of additive and/or synergistic effect of mAb-antibiotic cocktails. Antibody engineering is greatly progressing thanks to the pioneering work in cancer research which is now being applied to infectious diseases as well. For example, a hexamerization method alternative to the Hexabody technology and based on the IgM tail-piece has been reported [156]; an IgG1/IgG3 chimeric antibody was described as endowed with enhanced cytotoxic activity [157]; and bi- and tri-specific antibodies were produced to expand the breadth of reactivity [158,159,160]. Special attention is reserved for single-domain antibodies, also known as VHH (when they originate from camelids) or nanobodies, which are simpler molecules as compared to full-length human antibodies. *A. baumannii*-specific nanobodies have been identified [161], as well as anti-*Shigella* IpaD VHHs, which inhibit the activity of the Type 3 Secretion System [162]. Investigation of IgA functional properties in promoting enchained bacterial growth [163] is paralleled by the design of efficient expression and purification procedures [164]. Most recently, the delivery of mAbs as mRNA molecules was validated for an antibody against chikungunya infection [165], while detailed studies of mAb glycosylation profiles prompted mAb glycoengineering projects with the purpose of creating new molecules that can modulate the host’s immune system to tackle intracellular pathogens [166].

Of note, currently, mAb production relies on well-established protocols that do not require chemical synthesis and allow easy scale-up, although associated costs still represent an issue, especially if these medications are intended for use in LMICs. Constant effort should be directed to promote the availability of both therapeutic and prophylactic approaches to emerging countries where AMR is a major public health problem as well.

Overall, vaccines and mAbs represent two valid complementary approaches to fight AMR. Synergies between the two technologies have emerged: potent human mAbs can be isolated from vaccinated subjects or patients naturally exposed to a certain pathogen, and this can help understand the mode of action of the vaccine, identify novel antigens for vaccine development and determine antibody functionalities correlating with protection.

## 7. Conclusions

In conclusion, AMR represents one of the most critical threats to global public health and the scientific community is committed to fighting this challenge by developing new alternative therapies to traditional antibiotics. Vaccines and mAbs constitute complementary valid approaches to this scope. Advancements in the recent years have made innovative technologies available in both areas. A better understanding of their general applicability to AMR pathogens and mode of action will help accelerate the development and implementation of novel interventions. It will also be critical to document and quantify the impact that vaccination and the use of mAbs can have on antibiotic use and the spread of AMR to prove their value and support further research in the field. Additional research in the field is needed to identify optimal antigens to target some pathogens, better understand the immunology behind some infections still difficult to treat, increase the platformability of innovative technologies, establish clinically relevant in vitro and in vivo models and accelerate testing of novel candidates in small size human trials to reduce the probability of failure in larger and longer clinical trials after spending years with preclinical studies.

## Figures and Tables

**Table 1:** AMR pathogens, according to the WHO prioritization list, associated types of antibiotic resistance and preventive/treatment options under development over the last five years.

Bacteria	Type of Antibiotic Resistance [8]	Vaccines under Development ^§^	mAbs under Development	References
Priority 1: Critical ^#^				
*Acinetobacter baumannii*	carbapenem resistant		Murine anti-capsular mAbs (preclinical)	[13,14]
*Pseudomonas aeruginosa*	carbapenem resistant		WVDC-5244 (preclinical) anti-PcrV mAb (preclinical) MEDI3902 (MedImmune Astra Zeneca, Ph2) anti-SpuE mAb (preclinical)	[15,16,17,18,19]
*Enterobacteriaceae* *	carbapenem-resistant, 3rd-generation cephalosporin resistant	ExPEC 9V (J&J-Sanofi), bioconjugate, Ph3; FimHC (Sequoia), subunit vaccine, Ph2; Kleb4V (LMTB-GSK), bioconjugate, Ph2	Anti-O-Antigen and anti-capsule K. pneumoniae mAbs, preclinical Secretory IgA vs. enterotoxigenic *E. coli*, preclinical	[20,21,22,23,24,25] (NCT06134804; NCT04959344)
**Priority 2: High**				
*Enterococcus faecium*	vancomycin resistant		Murine anti-capsular and anti-secreted antigen A mAbs, preclinical	[26]
*Staphylococcus aureus*	methicillin-resistant, vancomycin intermediate and resistant	Staph 5V (GSK), subunit vaccine, Ph2	MEDI4893 (anti-a toxin) (MedImmune Astra Zeneca), Ph2	[27,28]
*Helicobacter pylori*	clarithromycin resistant			
*Campylobacter jejuni*	fluoroquinolone resistant		Anti-FliD secretory IgA, preclinical	[29]
*Salmonella* spp.	fluoroquinolone resistant	Entervax (ZH9PA+ ZH9), (Prokarion), live attenuated, Ph1; O:2-TT+Vi-TT (NIH, Lanzhou), glycoconjugate, Ph2; O:2-DT+Vi-TT (SII), glycoconjugate, Ph1; O:2-CRM+Vi-CRM (GSK/BioE), glycoconjugate, Ph1; INTS-TCV (GSK), GMMA/glycoconjugate, Ph2; iNTS COPS-FliC + TypBar (TCV) (Maryland U, Bharat), glycoconjugate, Ph2	Murine anti-outer membrane protein mAb Sal-06, preclinical Anti-Type 3 Secretion System mAb, preclinical Anti-LPS Sal4 IgA, preclinical	[30,31,32,33]
*Neisseria gonorrhoeae*	3rd-generation cephalosporin resistant, fluoroquinolone resistant	NgG (GSK), GMMA, Ph2	2C7 (anti-lipooligosaccharide mAb), preclinical	[34,35,36] (NCT05630859)
**Priority 3: Medium**				
*Streptococcus pneumoniae*	penicillin non-susceptible	Pn-MAPS 24v (GSK), MAPS, Ph2; Vax-24 (Vaxcyte), glycoconjugate, Ph2	Anti-capsular mAbs, preclinical	[37] (NCT05844423)
*Haemophilus influenzae*	ampicillin resistant	na **	Anti-Type 4 pilus mAb, preclinical	[38]
*Shigella* spp.	fluoroquinolone resistant	ZF0901 (Beijing Zhifei), glycoconjugate, Ph3; S4V-EPA (LMTB), bioconjugate, Ph2; altSonflex1-2-3 (GSK), GMMA, Ph2; SF2a-TT15 (Institute Pasteur), synthetic conjugate, Ph2; InvaplexAR-DETOX (Walter Reed), subunit, Ph1; ShigOravax (Hilleman Lab), killed, Ph1	Anti-Type 3 Secretion System mAb, preclinical	[32,39]

^#^ This classification follows that from WHO on antibiotic-resistant pathogens, published in 2017 [8]. Mycobacteria (including *Mycobacterium tuberculosis*, the cause of human tuberculosis) were not subjected to review for inclusion in the WHO prioritization exercise, as it was already considered a globally established priority for which innovative new treatments are urgently needed. * *Enterobacteriaceae* include *Klebsiella pneumoniae*, *Escherichia coli*, *Enterobacter* spp., *Serratia* spp., *Proteus* spp., *Providencia* spp. and *Morganella* spp. ^§^ Only vaccines in clinical development are listed. ** Vaccines containing Hib conjugates are available in the market.

**Table 2:** Advantages and disadvantages associated with antibiotics, monoclonal antibodies and vaccines.

	Advantages	Disadvantages
**Antibiotics**	Immediate effectiveness soon after administration, lifesaving during acute bacterial infections.	Selection of escape mutants, short-term activity, possible side effects.
**Monoclonal Antibodies (mAbs)**	Safety, high target-specificity, less susceptible to resistance mechanisms, longer half-lives (~21 days for IgG) compared to antibiotics.	High production cost, possible need for mAb cocktails, problems in reaching the target antigen due to biofilm formation or presence of capsule.
**Vaccines**	Multiple antigens can be targeted, low risk of developing resistance, preventive action (prophylaxis), long-lasting effectiveness, induction of immunological memory, herd immunity.	2–3 weeks required to elicit an immune response, full protection can often require multiple doses.

## Data Availability

Not applicable.
